# The AgeGuess database, an open online resource on chronological and perceived ages of people aged 5–100

**DOI:** 10.1038/s41597-019-0245-9

**Published:** 2019-10-31

**Authors:** Julia A. Barthold Jones, Ulrik W. Nash, Julien Vieillefont, Kaare Christensen, Dusan Misevic, Ulrich K. Steiner

**Affiliations:** 1 Department of Biology, University of Southern Denmark, Odense, Denmark; 2 Department of Marketing and Management, University of Southern Denmark, Odense, Denmark; 3 JV Conseil Internet Consulting, Saint Nom-la-Bretèche, France; 4 Department of Public Health, University of Southern Denmark, Odense, Denmark; 5 CRI - Center for Research and Interdisciplinary, Paris, France

**Keywords:** Biomarkers, Evolution

## Abstract

In many developed countries, human life expectancy has doubled over the last 180 years. Underlying this higher life expectancy is a change in how we age. Biomarkers of ageing are used to quantify changes in the aging process and to determine biological age. Perceived age is such a biomarker that correlates with biological age. Here we present a unique database rich with possibilities to study the human ageing process. Using perceived age enables us to collect large amounts of data on biological age through a citizen science project, where people upload facial pictures and guess the ages of other people at www.ageguess.org. The data on perceived age we present here span birth cohorts from the years 1877 to 2012. The database currently contains around 220,000 perceived age guesses. Almost 4500 citizen scientists from over 120 countries of origin have uploaded ~4700 facial photographs. Beyond studying the ageing process, the data present a wealth of possibilities to study how humans guess ages and who is better at guessing ages.

## Abstract (summary)

**Table:** 

Measurement(s)	Demographics • perceived age
Technology Type(s)	digital curation • computational modeling technique • Citizen Science crowd sourcing
Factor Type(s)	age • sex • ethnicity • birth country • age in photograph
Sample Characteristic - Organism	Homo sapiens
Sample Characteristic - Location	worldwide

Machine-accessible metadata file describing the reported data: 10.6084/m9.figshare.9934316

## Background & Summary

Record life expectancy among the world’s countries has risen steadily by 3 months per year for the last 180 years^1^. Underlying this remarkable extension is a change in how we age. With rising life expectancies and associated declines in fertility rates, senior citizens steadily increase as a proportion of the population in most developed countries—a phenomenon known as population ageing^2^. Population ageing heralds a suite of challenges for the economy, social security, and health care of countries^3,4^. Therefore, it is a pressing task to understand how the human ageing process has changed over the last 100 years and to predict how it will continue to change in future. Here we present a unique open-access database, rich with possibilities for studying ageing: the AgeGuess database on people’s perceived ages (i.e. how old someone looks to others) and chronological ages. The perceived age data originate from a citizen science project, where people upload pictures, mostly of themselves, to the webpage at www.ageguess.org and estimate the ages of other users.

Perceived age is an established biomarker of biological age. Biological age describes the relative condition of, for example, the cardiovascular, metabolic, or immune system. Biological ageing is therefore a change in functioning of these systems over time. It is usually determined by measuring an array of biomarkers of molecular and cellular events, which then are compared to a cohort average to determine biological age^5^, but biomarkers of ageing are as complex as the biological phenomenon itself^6,7^. Perceived age emerges as an excellent candidate biomarker for biological age that correlates with many cognitive and physical functions and has been shown to even predict mortality hazard for older people to a larger degree than chronological age^8–12^. Using perceived age as a biomarker for biological age gives ageing researchers a low-cost tool to widen their traditional focus from the oldest-old to studying how biological age already varies among young adults^10^. Furthermore, photographs of the same person at different ages allows to study within-individual progression of ageing, while older photographs open up possibilities to study how the relationship between chronological and perceived age shifts over time. Is a 40-year-old today biologically younger than a 40-year-old in the 1990’s?

How old we look to others does not only reveal our biological age, it is also part of our identity and plays a role in how people interact with us. Accordingly, perceived age is of interest to diverse disciplines, including psychology, forensics, and computer science. Age as a biometric measure to identify persons plays a role in the search of fugitives or other missing persons, for many of whom images must be corrected for age progression of the individual. Computer-aided ageing of facial images, and the estimation of age of persons by computer vision, are salient tasks for computer scientists^13–15^. Psychologists study the cognition of estimating ages and are joined by sociologists in asking how age affects human interactions and structures societies^16–19^. While the AgeGuess database was initiated by ageing researchers, it may hold answers to questions posed in other fields.

**Fig. 1:**
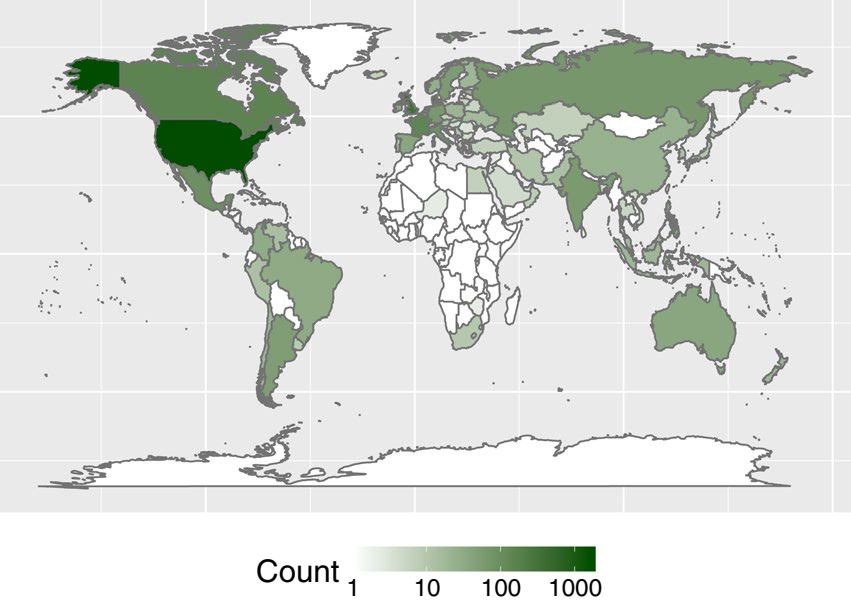
AgeGuess users by country of origin (n = 4434).

**Fig. 2:**
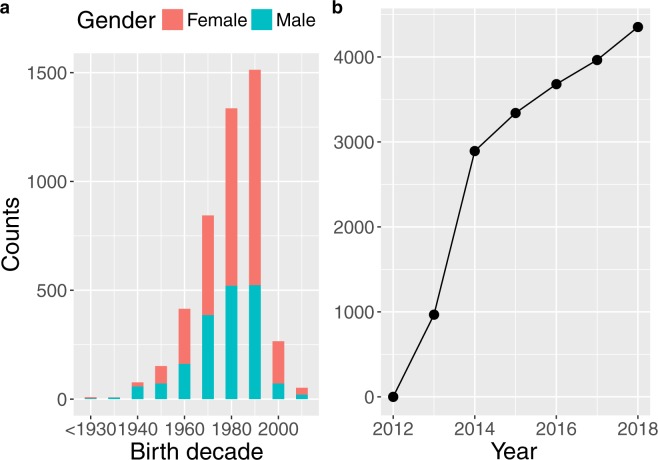
Summary of perceived age data in the AgeGuess database. (**a**) Number of photographs by sex and birth decade of individual (n = 4710). (**b**) Cumulative numbers of users over study year (n = 4434).

Here we present a wealth of perceived age data spanning birth cohorts from the years 1877 to 2012. The data were collected by a citizen science project since 2012, where citizens upload facial pictures of individuals with known age and guess the ages of faces on images uploaded by other users. The data collection is ongoing. Almost 4500 citizen scientists from over 120 countries of origin have uploaded ~4,700 pictures (Figs 1, 2a). The citizen science project continues to grow steadily (Fig. 2b). Communication by the media and outreach activities on social media (Facebook: www.facebook.com/ageguess.org/, Twitter: @ageguess_org) and in person (e.g. open science days) aim to both recruit more users and to inform the public about the change in how we age.

In the following, we introduce how we collect the data via a webpage and how we recruit citizen scientists to participate in the project. We describe the database in detail, providing both a summary of the data and the information on the database variables. Finally, we critically reflect on the data quality showing that the quality of data collected by the citizen scientists equals that of more controlled studies. We end by suggesting areas of research that could exploit the database.

## Methods

### The webpage

#### Organisation

The webpage at www.ageguess.org is the platform we use to collect the perceived age data. The webpage is licensed under a Creative Commons Attribution-NonCommercial-NoDerivatives4.0 International License. The webpage is hosted in France and has been approved by the Commission nationale de l’informatique et des libertés (CNIL, National Commission on Informatics and Liberty; declaration #1800944v0), France’s regulatory body ensuring the application of data privacy law to the collection, storage, and use of personal data. The webpage is built with Drupal (www.drupal.org), a free and open-source content management system (CMS) based on PHP and MySQL. Drupal provides login/logout functionalities and account activation and deletion. While users retain the copyright of their pictures, the AgeGuess project owns the rights to use content on the webpage covered by intellectual property rights, including text, images, graphics, logos, icons, sounds, and software (see also Rules of AgeGuess and the consent given when creating an account). The full terms and conditions are available at www.ageguess.org/legal-notice. Below we describe the most prominent features of the webpage regarding data collection. Further details about both the front and back-end structure and behaviour of the webpage can be received upon request.

#### Data collection

To contribute to the data collection, users create an account with a verified email address, link to or upload photographs of themselves and others, and provide basic information about themselves—or others on their uploaded photographs respectively: birth year, age in the photograph, ethnicity, and birth country. (Note that “ethnicity” might be denoted more accurately by “race” to reflect shared physical features and not cultural ones, but we keep using the term “ethnicity” to guarantee coherence in the data collection). They can then proceed to guess the ages of other users in these users’ photographs. After each guess, the users see the real age of the person in the picture, summary statistics of previous guesses of other users, and a histogram of previous age guesses. Similarly, the user can see this information for their own photographs on the user’s personal account page. As an additional feature, users can also upload photographs of other people (e.g. relatives) for which the user owns the copyright or that are available under Creative Commons license. The users provide the same basic information for the persons in the photographs as they provided for their own photographs and specify their relationship (i.e. friend or family member). The minimum legal age to open an account is 14 years, but pictures uploaded can show people of younger age.

To provide a further incentive to contribute, the webpage is set up as a simple online game. For uploading pictures and for each guess, the users receive a number of points depending on the accuracy of the guess (exact guess 10 points, 1–2 years off 7 points, 3–5 years off 5 points, 6–10 years off 2 points, more than 10 years off 1 point, 10 points for each uploaded or linked picture). On their personal account page, the user can see the cumulative number of gained points, the number of guesses made, and the proportion of fully accurate guesses. At www.ageguess.org/ranking users can compare how good they are at guessing ages in comparison to other users by point scores and by the mean deviation of their age guesses from the real age of displayed persons. Deviations arise both due to the guesser’s uncertainty and due to actual deviations of biological ages from real ages. Users can locate their own position on ranking lists by clicking a provided button.

The webpage displays photographs to users for guessing following a specific algorithm, which creates a queue, an ordered list of photos that will be shown to users to guess. To be eligible to be picked by the algorithm for display to a user, pictures have to fulfil the following criteria: (1) not be uploaded by the user themselves to prevent users rating their own pictures, (2) not being guessed and/or seen already by the user, and (3) not being reported more than four times. Users can report pictures when making guesses by clicking one of the options: rotation needed, cropping needed, edited image, missing person, more than one person, copyright infringement, and offensive content such as nudity or violence. Considering these criteria, the algorithm sorts the pictures ascendingly by number of previous guesses, placing photos with fewer guesses at the top of the list. Pictures with the same number of guesses are chosen at random. Furthermore, the user can skip a photo via a “skip” button, for example to avoid guessing ages of people the user knows. The user can however not skip more than 4 times per session. The system renews the queue each time a user logs in.

It is worth noting that features of the algorithm and the overall system were gradually refined over time with growing knowledge on users’ behaviour and potential problems. A suite of countermeasures against malicious users are in place. For example, to reduce the number of malicious users, who may upload unauthorised pictures, try to get access to pictures of other users, and/or troll the webpage operations, the webpage requests email validation at registration. We do not share the full details of these measures in order to keep malicious users at a disadvantage.

#### Obtaining the AgeGuess data

Users with an active AgeGuess account can download the data described in the next section from a repository at www.ageguess.org/download. The data for download are directly extracted from a MySQL database, so users have continuously access to the most up-to-date data. Furthermore, a data version dating from spring 2019 is available for download from the UK Data Service^20^. The data are released under a Creative Commons Attribution-NonCommercial-ShareAlike 4.0 International license (CC BY-NC-SA 4.0). For attribution, we urge users to cite this paper when using the data. All data are fully anonymised and any attempt to reveal the identity of users violates AgeGuess terms of use. We do not publish the photographs open-access to respect EU privacy protection law. While we cannot make the photographs widely available, we are able to make bilateral agreements with individual researchers or groups to ensure the use of the photographs is in line with the consent obtained. This will involve drawing up a formal data sharing or collaboration agreement. The nature of the agreement depends on the jurisdiction and properties of the legal entity who enters the agreement and will be finalised by the legal support of the AgeGuess project. Interested parties can send a request using the contact form at www.ageguess.org/contact or send an e-mail to contact@ageguess.org. Sharing of the photographs depends solely on a legal agreement to ensure the use of the photographs is in line with the consent obtained. We do not place any conditions on the re-use of the photographs with respect to competitive re-analysis or to ensure special authorship rights for the data generators.

## Data Records

### History and internal organisation

In 2012, U.K. Steiner and D. Misevic started the AgeGuess.org citizen science project. They form the core committee and are responsible for creating and updating protocols for data collection and for the overall infrastructure of the database, as well as for securing funding. They are supported by webpage building and database expert, J. Vieillefont, who created and maintains the current version of the webpage and the database. The first fully functional version of AgeGuess.org was coded by Charlotte Le Pesquer. Furthermore, a team of scientific advisors spanning both academic disciplines (e.g. public health) and industry (e.g. pension providers) helps shape the scientific directions of the project and highlights funding opportunities. Depending on availability of funding, one or more pre- or post-doctoral fellows have worked on data analysis and outreach.

### Variables and descriptions

The most up-to-date version of the data is accessible to the public (i.e. users with an user account at ageguess,org) as five csv files; for those who do not have an account and do not want to create one a version dating from spring 2019 is available for download from the UK Data Service^20^. The data that is continuously and ongoing collected are stored in a MySQL database. These five csv data files respectively contain information on *guess*, *photos*, *gamers*, *quality*, and *report*, using those names with the prefix “*ag_*” for AgeGuess and .csv extensions. In the following, we describe the variables in each of the csv files. All missing data are encoded as NA.

The *ag_guess*.*csv* file stores the information regarding the age guesses using the following variables: *uid*, *guess_id*, *photo_id*, *ageG*, *outG*, and *access*. The *uid*, *guess_id*, and *photo_id* variables contain the individual identifiers of the user who made the guess, the guess itself, and the photograph guessed on. The *ageG* and *outG* variables describe the guessed age and the deviation in the guess from the real age in years, respectively. The access variables store the timestamp when the guess was made in date and time UTC + 1:00 in the format ‘YYYY-MM-DD HH:MM:SS’. While repeated guessing by the same person on the same photograph is no longer possible due to the current version of the algorithm controlling the photos displayed to the users, this was possible in early implementations of AgeGuess. Data on repeated guesses are available from previous versions of the database upon request.

The *ag_photos*.*csv* file stores the information regarding the photographs using the following variables: *uid*, *photo_id*, *age*, *relation*, *gender*, *ethnicity*, *birth_country*, *birth_year*, *death_age*, and *created*. The *uid* and *photo_id* variables represent the individual identifiers the user who uploaded the photograph and of the photograph. The *relation* variable indicates whether the photograph is of the user or of another person to which the user has a relation (categories: user, unrelated of friend, mother/father, son/daughter, sibling, half sibling, maternal/paternal grandparent, maternal/paternal aunt/uncle, maternal/paternal cousin, grandchild). The *gender*, *ethnicity*, *birth_country*, *birth_year*, *death_age* variables contain the respective basic demographic information for the person in the photograph. The *created* variable stores the timestamp when the photograph was added in date and time UTC + 1:00 in the format ‘YYYY-MM-DD HH:MM:SS’.

The *ag_gamers*.*csv* file stores the information regarding the users (aka gamers) with the following variables: *uid*, *g*, *ng*, *points*, *gender*, *ethnicity*, *birth_country*, *birth_year*, *access*, and *created*. These variables store the individual identifier of the user (*uid*), the number of correct guesses the user made (*g*), the number of other guesses (*ng*), and the points gained in the online game (*points*). Furthermore, the file contains the users’ basic demographic information regarding gender, ethnicity, birth country, and birth year, stored in variables of these names. Finally, the *access* and *created* variables store the timestamp in date and time UTC + 1:00 of when the user last logged in and of when the user created an account with AgeGuess, respectively.

The *ag_quality*.*csv* file contains information on quality reports that users have made on photographs. The variables are *uid*, *quality_id*, *photo_id*, *quality*, and *created*. The *uid*, *quality_id*, and *photo_id* variables contain the individual identifier of the user who made the assessment, the identifier of the quality assessment, and of the photo on which the assessment was made, respectively. Quality itself is encoded as 1 = high, 2 = medium, 3 = low in the *quality* variable. The timestamps of the assessment in formats described above are stored in the *created* variables.

Finally, the *ag_report*.*csv* file pertains to information on any other reports made on photographs. The variables are *uid*, *photo_id*, *report_id*, *comment*, and *created*. The *uid*, *photo_id*, *and report_id* variables store the individual identifiers of the user who made the report, the photograph on which the report was made, and the report itself, respectively. Report categories are *rotation needed*, *cropping needed*, *none or more than one person*, *copyright infringement*, *offensive content*, and combinations thereof. The AgeGuess team regularly edits photographs after receiving a report, for example when cropping is needed, and retains the edited photographs if suitable. Photographs and data associated to the other report categories are deleted. Finally, after internal checks the system adds reports related to missing photographs and inaccurate data on birth year and age. The timestamps of the report in formats described above are stored in the *created* variables. The *ag_quality*.*csv* and *ag_report*.*csv* are mostly for system-intern use and the data are not included in the distribution at www.ageguess.org/download but can easily be received on request.

### Data summary

After running the data cleaning protocol (see below), AgeGuess has, as of spring 2019, 4434 users from ~120 countries of origin of which 2339 are female, 1757 male, and the rest is unknown (Fig. 1). Most users identified as Caucasian/White (3024), followed by Asian (299), Hispanic (265), Black (120), Other (208), and 518 users did not provide an answer. The users have uploaded 4710 photos of 2855 females and 1855 males (Fig. 2). The age of the persons displayed in the photographs ranges from 5 to 100 years old. The earliest and latest corresponding birth years were 1877 and 2012, respectively. The persons in the photos were identified as Caucasian/White (3746), followed by Asian (343), Hispanic (255), Black (103), and Other (246). The data contain repeated measures on 519 individuals with more than 242 individuals having uploaded three or more pictures of themselves.

**Fig. 3:**
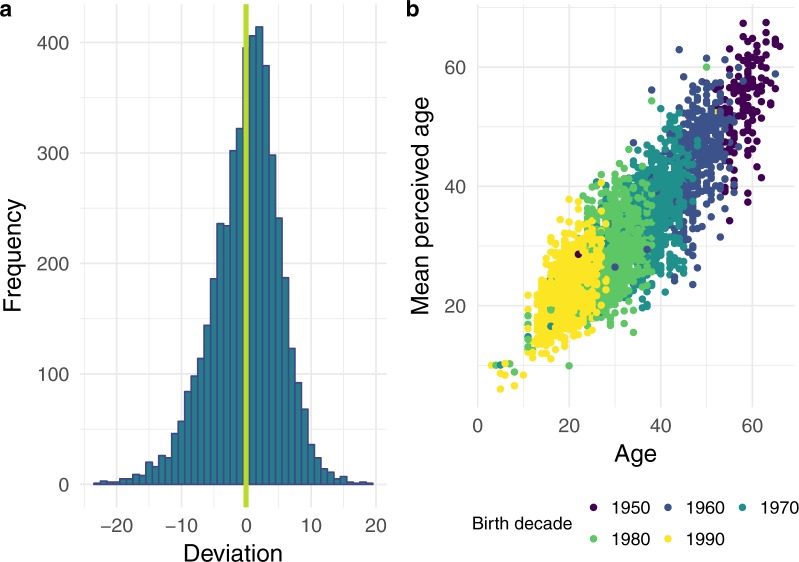
(**a**) Frequency distribution of the deviation of the mean guessed age from real age (n = 4710). The green line marks no deviation between mean perceived and real age. (**b**) Mean perceived age plotted against real age. Each data point represents the mean perceived age of one of the 4710 images. Data points belonging to different birth decades are coloured differently.

Overall users have guessed ages 220,231times. We have at least 10 repeated guesses for each photograph, with a maximum of 385 repeated guesses and a median of 42 guesses. The variation in number of guesses stems from earlier versions of the photograph-selecting algorithm, which did not account for the number of previous guesses on a photograph. The deviation of the mean age guess from real age for each photograph is normally distributed with a mean close to 0 (Fig. 3a). The relationship between mean perceived age and real age for each photograph is shown in Fig. 3b.

### Technical validation

The data originate from citizen scientists. Such data are often approached with skepticism from the scientific community, even though citizen scientist frequently perform equally well as trained scientists in collecting data^21^. The data collected can contain both false and missing data that may have been entered by users either by mistake or intentionally. Therefore, we perform some basic data cleaning steps before publication of the data and provide basic tests for data quality and accuracy. From the *Guess* data we delete all guesses that are more than two times the standard deviation away from the mean age guess on a photograph. We further remove all guesses on photographs that have less than 10 guesses, since it is known that substantial uncertainty in rating ages exists^19,22,23^. This uncertainty mainly arises within guessers among repeated guesses and to a much lower degree among guessers^24^. Simply put, a perceived age estimate based on only a few guesses is less accurate than one based on more than 10 guesses, and therefore, we exclude photos with less than 10 guesses to improve data quality. Using the information in the *Report* data (see above), we delete guesses on photos with inaccurate age or birth year. Since not all inaccurate birth years are flagged by internal system checks, we replace in both the *Photos* and *Gamers* data all unrealistic birth years (<1800 or >2019) with NA. The whole, uncleaned data set can be obtained upon request.

Furthermore, the data quality of the AgeGuess database is subject to a trade-off common to many citizen science projects, where large quantities of data are obtained at the expense of representativeness of sample and data accuracy. Neither the AgeGuess users nor the persons displayed in the photograph are representative samples of the population with respect to age, geographic location, or ethnicity, though information on both the displayed person in the images and the users is available to account for biases. Such biases are also frequently found in classic scientific studies^8,25,26^. Furthermore, the uploaded photographs are not standardised with respect to posture, lighting, face expression, clothing, background, distance to camera, hairstyle or dye, make-up, or the use of accessories such as hats, jewellery, or glasses. Some of these factors may be used to deliberately conceal age: older adults may use particularly make-up and hair dye to appear younger, while younger adults may manipulate their looks to appear older. A bias for older individuals being perceived younger and younger individuals being perceived older has been shown to be independent of such factors^24,27^. We do discourage editing photographs to alter the age appearance and offer a report option to flag those photographs, however, some manipulated photographs may have remained unnoticed.

We have no direct means to control the accuracy of the chronological age users enter when uploading photographs. However, we can indirectly detect large mistakes or deliberately provided false information by identifying and excluding outliers. In certain citizen science projects concerns arise due to the ability of the citizen scientists to accurately perform the demanded tasks compared to classical trained scientists. For the database presented here this should be of little concern. Previous highly controlled scientific studies on perceived age rating showed that geriatric nurses, who were considered experts in rating ages of older women, did not perform different in rating ages compared to two other groups: young male students, who were expected to be the worst raters, and same-aged peers, i.e. older women^8^. Confidence in the collected data also comes from small side-projects that allow us to assess the quality of the data. For instance, 10 students at the University of Southern Denmark aimed at outcompeting the users of AgeGuess, first by spending several weeks studying scientific literature on factors that influence perceived age to train themselves to be good at rating ages. When they rated ages on AgeGuess.org, they were disappointed to not have performed any different than the users on AgeGuess.org (data not published). Also, when comparing the variance (standard deviation, SD) in the difference between chronological age and perceived age between highly controlled studies^19,22–24^ and the AgeGuess data the variance in the difference was comparative to the data generated by the citizen scientists (6–8 in classical studies, 6.9 for the AgeGuess data). This similarity in age estimation might not be expected since the images in the controlled studies have been obtained under strict standardized settings, such as controlled posture, lighting, face expression, clothing, background, distance to camera, make-up, and without accessories such as hats, jewellery, or glasses^8^. Such standard settings should lower variance. The classical studies partly included specific age groups, e.g. some have included only persons above 70^8^, and variance is increased for judging the age of older individuals^23^, as is also found in our data where the variance at least of the oldest old is slightly higher compared to very young persons guessed on (Fig. 3b). A limited age range can also reduce the variance since the raters realize that the persons guessed on are within such an age range^22^.

Overall, the citizen science data agrees with basic findings of controlled studies and shows similar variances. Still, anyone using the data should be aware of the uncertainties that come with a citizen science approach of collecting data and that such data is prone to additional error and noise, even though we could not yet detect such increased error. We therefore evaluate the data collected by the citizen scientists to be largely accurate and that the quantity of the data (guesses made) vastly outweighs the potential data quality issues, such as missing data and data entered by mistake or intentionally erroneously entered.

## Research Opportunities

The AgeGuess data provide exceptional opportunities to approach research questions across scientific fields. The research opportunities broadly split into two main directions. The first one relates to ageing research and some of the questions outlined in the introduction, e.g. evaluating ageing processes by studying temporal variation in biological age and the difference between perceived and chronological age. The second direction relates to a more sociological view, where the users themselves are the subject of study, and research questions center around their ability to guess ages.

Regarding the first research direction, the collected data on perceived age and chronological age can inform on the basic ageing process. The data can reveal whether more recent birth cohorts are biologically younger than their earlier counterparts, e.g. is a 40-year-old today biologically younger than a 40-year-old in the 1980s, and does that difference hold in the same way for 30-year-olds? We can investigate how shifts in biological age over time accompany shifts in life expectancy^10,24^. Research questions can also focus on how ageing happens within a lifetime, i.e. do we age continuously throughout our lives or are there boosts and arrests of ageing? Furthermore, quantitative geneticists can exploit relatedness among persons in uploaded photographs to study diverse questions, including to what degree biological age is heritable, and how heritability of biological age relates to known heritability of lifespan. The number of images is not vast, but computer scientists can use the data to train and evaluate age estimation algorithms^28,29^. Such training and exploration could go beyond the fast developing and advancing artificial intelligence (AI) approaches that aim at a most accurate age estimation of an image. Many of the currently used databases for such training of AI networks do not include data on perceived ages and AI might use very different signals than us humans to evaluate the age. However, as outlined above the deviation between real age and perceived age is informative as a biomarker of ageing. AI would not be able to detect shifts in human age perception over time that might reflect adjustment to different biological ages^24^. Various of the databases that are used to train AI networks suffer from biases, e.g. images of celebrities that might not be representative^28,30^. Access to the original pictures of AgeGuess requires a close collaboration with the AgeGuess project due to new EU regulations on privacy of identifiable pictures of persons.

The second research direction centers around the users and their ability to guess ages. General questions about the ability to guess the age of people include: (1) are we better at guessing the age of our own age group, (2) does our ability to guess ages increase with experience (age or number of previous guesses on AgeGuess), and (3) are we better at guessing the age of our own sex or the opposite sex, and is the age of one sex easier to guess than the other? Such questions can test hypotheses derived from sexual selection theory, for example in the context of human partner choice. Furthermore, studying whether it is easier to guess the age of people of our own ethnicity compared to other ethnicities might provide information on generalities of ageing processes and commonalities of ageing signs. The data might furthermore reveal guessing abilities related to exposure to specific ethnic or age groups. For example, contrary to expectation geriatric nurses, who daily work with elderly patients, are not better at guessing ages of elderly citizens than male students^8^.

As focus turns from the subject in the photograph to the judgment, the AgeGuess data become highly valuable for answering basic questions about information processing. More specifically, the data provide an outstanding opportunity to investigate to what extent can the hidden aspects of the environment be measured using perceptible cues that correlate more or less with these unobservable parts. This problem of inferring hidden aspects is, and has been, fundamental to human survival, and is therefore important for cognitive psychologists and neuroscientists alike^31^. Moreover, in their development of AI, the cue learning problem is, with some controversy, being studied by computer scientists^32^. How much information about a person can be extracted from the person’s facial features, and to what extent it matters how different features of the face are combined in making the judgment^33^ are certainly interesting questions. They are also questions with potential to challenge the organisation of a society in which judgments are done at mass scale by computers with live access to extensive networks of cameras.

Here we present a unique database rich with possibilities to study the human ageing process: the AgeGuess database on people’s perceived and chronological ages. The database comprises >220,000 perceived age guesses. The questions that we discuss are certainly not exhaustive and are meant only as an illustration of the power of the data for answering broad scientific questions. We believe many more questions can be addressed using the AgeGuess data, including those that we as data collection initiators have not thought about and may not be able to imagine. We thus provide open access to the data free of charge, invite researchers and citizens alike to tap into this rich resource, and look forward to seeing interesting and unexpected results and outcomes.

## Data Availability

All code for the data presentation and processing has been done in program R 3.6.0 and can be obtained upon request.
